# Uptake and Processing of the Cytolethal Distending Toxin by Mammalian Cells

**DOI:** 10.3390/toxins6113098

**Published:** 2014-10-31

**Authors:** Joseph M. DiRienzo

**Affiliations:** Department of Microbiology, School of Dental Medicine, University of Pennsylvania, 240 South 40th Street, Philadelphia, PA 19104, USA; E-Mail: dirienzo@pobox.upenn.edu; Tel.: +1-215-898-8238; Fax: +1-215-898-8385

**Keywords:** AB toxin, *Aggregatibacter actinomycetemcomitans*, cytolethal distending toxin, endocytosis, endoplasmic reticulum, Golgi, nuclear localization signal, retrograde transport

## Abstract

The cytolethal distending toxin (Cdt) is a heterotrimeric holotoxin produced by a diverse group of Gram-negative pathogenic bacteria. The Cdts expressed by the members of this group comprise a subclass of the AB toxin superfamily. Some AB toxins have hijacked the retrograde transport pathway, carried out by the Golgi apparatus and endoplasmic reticulum (ER), to translocate to cytosolic targets. Those toxins have been used as tools to decipher the roles of the Golgi and ER in intracellular transport and to develop medically useful delivery reagents. In comparison to the other AB toxins, the Cdt exhibits unique properties, such as translocation to the nucleus, that present specific challenges in understanding the precise molecular details of the trafficking pathway in mammalian cells. The purpose of this review is to present current information about the mechanisms of uptake and translocation of the Cdt in relation to standard concepts of endocytosis and retrograde transport. Studies of the Cdt intoxication process to date have led to the discovery of new translocation pathways and components and most likely will continue to reveal unknown features about the mechanisms by which bacterial proteins target the mammalian cell nucleus. Insight gained from these studies has the potential to contribute to the development of novel therapeutic strategies.

## 1. Introduction

The cytolethal distending toxin (Cdt) is a subclass of the AB toxin superfamily. Typical members of this subclass are produced by Gram-negative bacteria and those that are expressed from a complete *cdt* operon have an AB_2_ subunit configuration. Representative examples include: *Aggregatibacter actinomycetemcomitans* (*Aa*Cdt); *Campylobacter* *jejuni* (*Cj*Cdt); *Escherichia coli* (*Ec*ICdt, *Ec*IICdt, *Ec*IIICdt, *Ec*VCdt); *Haemophilius ducreyi* (*Hd*Cdt); *Helicobacter* *hepaticus* (*Hh*Cdt); *Providencia alcalifaciens* (*Pa*Cdt); *Shigella boydii* (*Sb*Cdt) and *Shigella dysenteriae* (*Sd*Cdt) (reviewed in [1]). The A subunit, CdtB, is an enzyme, with an average molecular size of 29 kDa, that exhibits cation-dependent metalloenzyme activities, *in vitro*, characteristic of endonucleases [2,3], inositol polyphosphate 5-phosphatases [4] and sphingomelinases [5]. The B component is composed of two heterogeneous subunits, CdtA (23 kDa) and CdtC (21 kDa), that appear to participate cooperatively in binding of the holotoxin to the target cell surface. The CdtC subunit may also facilitate the entry of CdtB into the cell during endocytosis [6]. Several *Salmonella enterica* serovars, Typhi (*Se*tCdt) and Javiana (*Se*jCdt), carry only the *cdtB* gene and employ alternative strategies to intoxicate susceptible target cells [7,8,9]. CdtB makes its way to the nucleus by an endoplasmic reticulum-associated degradation (ERAD) or non-ERAD pathway followed by translocation across the nuclear membrane. In most cases in infected cell cultures, CdtB introduces double-strand breaks in the target cell DNA that activates cell cycle checkpoints leading to growth arrest and eventual cell death.

Significant strides have been made, over the last several decades, in understanding the inhibitory effects of the Cdt group of toxins on the proliferation of various types of mammalian cells. However, the structural and biological complexity of the Cdt, compared to that of other AB toxins, has made it a challenge to elucidate the mechanistic details of transport and trafficking. This review strives to present current information and hypotheses about the synthesis of the Cdt and mechanism(s) of infection of susceptible target cells. AB toxins that travel from the cell membrane to the ER and the nucleus provide novel model systems that can be used to (i) study retrograde transport pathways, (ii) elucidate the regulation of protein trafficking and (iii) design strategies for the precise localized delivery of therapeutic agents.

## 2. Synthesis and Secretion of the Cdt

### 2.1. Sec-Dependent Secretion

**Figure 1:**
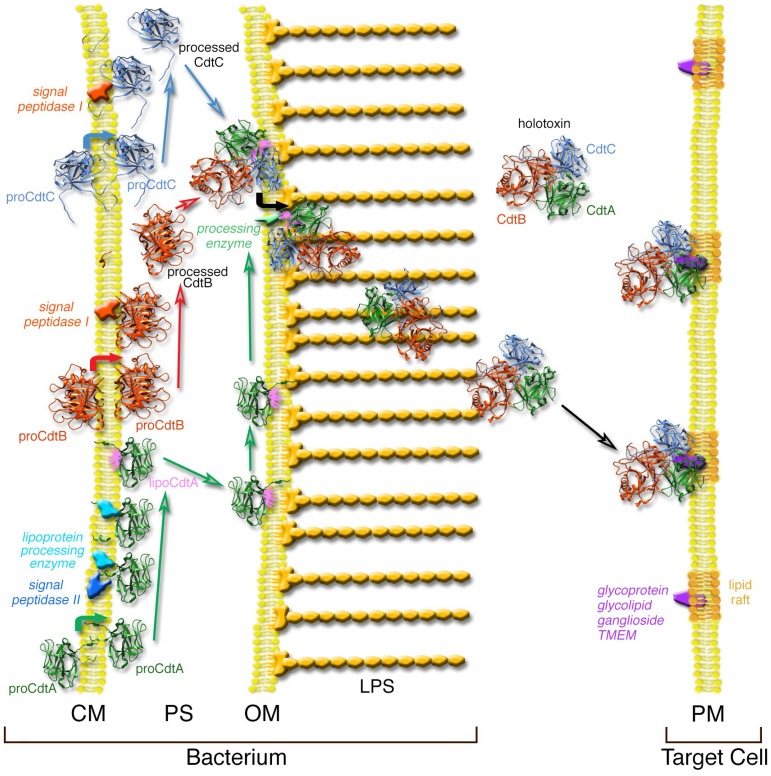
Model of *Aa*Cdt synthesis and secretion in *E. coli*. Each of the three subunits traverse the cytoplasmic membrane (CM) of *E. coli* and reach the periplasmic space (PS) after removal of a signal sequence. CdtA is modified with lipid that anchors the protein on the outer membrane (OM). The subunits self-assemble and the holotoxin is released from the membrane by removal of the lipid from CdtA. The holotoxin accumulates outside the cell where it is free to bind to the surface of susceptible cells in the environment presumably through a receptor-mediated process at the plasma membrane (PM). Details of the model are presented in the text. The peptidoglycan layer or bacterial cell wall, which is located in the PS, is not shown for clarity. LPS, lipopolysaccharide. Modified from a previously published model [10].

Much of our understanding of the biogenesis of the Cdt is based on the study of Ueno *et al.* [10] using the *Aacdt* genes cloned in *Escherichia coli*. The detailed steps in this process are summarized in Figure 1. The most interesting property of Cdt synthesis is that the subunits are assembled inside the cell (periplasmic space) and secreted as the biologically active holotoxin. Each of the three subunits is independently translocated across the cytoplasmic membrane due to the presence of a hydrophobic signal sequence. This is a standard mechanism for the movement of secreted proteins across the cytoplasmic membrane of bacteria [11]. Precursor proteins are exported by a common pathway that involves both signal peptidase I and signal peptidase II [12]. As CdtA exits the membrane it is modified with glycerolipid by a theoretical lipoprotein processing enzyme. *Aa*CdtA contains the lipid-binding motif (lipobox), LVAC, near the cleavage site for the signal sequence at the amino-terminal end of the protein. The signal sequence of the CdtA precursor protein is removed by signal peptidase II. The cleavage step releases the lipoCdtA from the cytoplasmic membrane and this hydrophobic subunit becomes anchored in the inner leaflet of the outer membrane (periplasmic space side). The amino-terminal signal sequences on both CdtB and CdtC precursor proteins are thought to be removed by signal peptidase I which is part of a *sec*-dependent pathway [13]. The processed CdtB and CdtC proteins accumulate in the periplasmic space. All three proteins self-assemble at the outer membrane. An unidentified processing enzyme removes the hydrophobic glycerolipid from lipoCdtA as the holotoxin is translocated across the outer membrane. An amino-terminal portion (approximately 6 kDa) of CdtA may also be removed along with the glycerolipid since a 17 kDa CdtA peptide (CdtA’) has been found in the cell-free spent medium fraction of *H. ducreyi* and *A. actinomycetemcomitans* [14,15]. The now hydrophilic holotoxin accumulates in the aqueous environment outside the bacterium until it recognizes a specific receptor on the target cell surface. There are several caveats to this model. The *Aa*Cdt is foreign to *E. coli*, therefore, signal sequences, signal peptidases and modifying enzymes may not be the same across species lines. Also, over-expression of genes can lead to the formation of inclusion bodies that would trap the gene products in the cytoplasm. To overcome this latter problem, a low copy number *E. coli* plasmid was used. Finally, parts of this process are still hypothetical and require further study.

### 2.2. Other Cdt Delivery Mechanisms

It has been suggested that the *Cj*Cdt and *Aa*Cdt holotoxins are released from the bacterium enclosed in outer membrane vesicles [16,17]. It was assumed that these vesicles contained assembled holotoxin complexes because all three toxin subunits were present, cells treated with the vesicles became distended and the cell cycle was arrested. A mechanism for delivering the toxin from the vesicles to the target cell was not postulated. However, one possibility is that the vesicles can fuse with the plasma membrane.

Although some studies have suggested that a CdtA-CdtB heterodimer is capable of intoxicating cells, the general consensus is that all three Cdt subunits are required, *in vivo*, to form a biologically active toxin that enters cells. However, *Salmonella enterica* serovars Typhi and Javiana contain only the *cdtB* gene [7,18]. This bacterium invades cells and multiplies in a *Salmonella*-containing vacuole (SCV) in macrophages [19,20]. CdtB may be delivered by the replicating bacteria when the membrane-enclosed SCV fuses with either the Golgi apparatus or ER. Interestingly, two pertussis toxin subunits, PltA and PltB, appear to form a complex with the *Se*tCdtB and may facilitate delivery of this active Cdt subunit through autocrine and paracrine cell communication pathways [7,8,9].

## 3. Steps in the Intoxication Process

### 3.1. Step 1—Recognition of Target Cells

As in the case of other AB toxins, the A chain or subunit that imparts biological activity to the holotoxin, must be translocated across the plasma membrane of the target cell. This is most likely a receptor-mediated process. However, unequivocal identification of a common receptor for the species-specific Cdts has been elusive. The first studies to address the identification of a Cdt receptor provided compelling evidence that the *Ec*IICdt and the *Aa*Cdt bound to a fucose-containing glyco-molecule and to ganglioside GM3, respectively [21,22]. Subsequent binding studies found that species-specific Cdts, such as *Ec*Cdt and *Hd*Cdt do not compete for binding to host cells [23]. These contrary findings were somewhat perplexing because of the thoroughness of these experiments. In the former study glycoproteins that contain N-linked fucose, such as fetuin and thyroglobulin, bound to *Ec*IICdtA and *Ec*IICdtC and blocked binding of the holotoxin to HeLa cells. Removal of N-linked sugars on the cell surface resulted in the loss of intoxication. In support of these results, it was found that the *Aa*CdtA strongly bound to N-linked fucose-containing glycoproteins *in vitro* [24]. Indeed, there is precedent for a lectin type of interaction among other AB toxins such as ricin. In contrast to glycoprotein structure, GM3 is a NeuAcα(2,3)-Galβ(1,4)Glc-ceramide. Mise *et al.* [22] showed that U937 cells (human monocyte cell line) treated with 1-phenyl-2-palmitoylamino-3-morpholino-1-propanol, a glucosylceramide synthesis inhibitor, and a cell line deficient in sphingolipid biosynthesis, were both resistant to inhibition by the *Aa*Cdt. Several other AB toxins bind to the gangliosides GM1 (cholera toxin), GD1a (pertussis toxin) or Gb3 (Shiga toxin). In another series of experiments using mutants of Chinese hamster ovary (CHO) cells deficient in glycolipids or a sialic acid galactose transporter no differences were found in the binding of the *Ec*ICdt or *Aa*Cdt or change in toxicity relative to that of the wild-type cells [25]. To further confound identification of a common Cdt receptor it was reported that the *Ec*ICdt recognizes the G-protein coupled receptor TMEM181 in a chronic myeloid leukemia cell line KBM7 [26]. TMEM181 mutants of this cell line were resistant to the toxin. In a modified screening procedure it was found that *Aa*Cdt does not require TMEM181 for intoxication but appears to be dependent on the integral membrane protein synaptogyrin 2 [27].

Only two of the species-specific Cdts, *Hd*Cdt and *Aa*Cdt, have been crystalized to date [28,29]. However, uniformity in subunit composition among the members of the Cdt group would support the existence of a relatively conserved holotoxin structure. In this structure a groove or pocket appears to reside between the CdtA and CdtC subunits. It has been hypothesized, based on an analogous comparison to ricin, that this groove is essential for toxin activity because it plays a role in receptor binding [28]. Another feature, identified by this group, important for receptor binding is the presence of a region composed primarily of surface-exposed heterocyclic and aromatic amino acids (tryptophan and tyrosine) on CdtA. It is curious that a number of tyrosines in this “aromatic patch” region of the *Aa*Cdt contributed to the binding of this subunit to fucose-containing glycoproteins *in vitro* [30]. The aromatic patch region, particularly residues W91, W98, W100, Y102 and Y141, is highly conserved among the various Cdts [31]. Therefore, it is reasonable to expect that the presence of a conserved groove and aromatic patch domain would suggest the existence of a common, or at least structurally related, Cdt receptor for the members of this group of toxins.

In addition to recognition of a specific receptor, a second important element for the binding of Cdt to cells appears to be involvement of a distinct area of the plasma membrane known as a lipid raft. Lipid rafts are self-organized parts of the lipid bilayer, enriched in sphingolipids, cholesterol and proteins, that represent subcompartments that serve as stabilized platforms for specific biological functions including membrane signaling and trafficking [32]. The *Aa*Cdt may bind to the plasma membrane in areas populated by lipid rafts based on the results of intoxication experiments using Jurkat cells [33,34]. It was postulated that binding localization was directed by a cholesterol recognition/interaction amino acid consensus (CRAC) sequence (^68^LIDYKGK^74^) in the *Aa*CdtC. The presence of a CRAC sequence in the *Aa*CdtC was first noted by Guerra and coworkers [35]. However, like the receptor controversy, there are mixed results in attempting to establish a requirement for lipid rafts in Cdt binding to susceptible cells. Cdt binding may be either cholesterol-dependent or independent based on (i) the specific cell type or cell line, (ii) the species-specific origin of the toxin, (iii) toxin concentration and (iv) reorganization of the putative receptor for toxin binding within protein components of the cell membrane [23,25]. Cholesterol depletion of cells is typically used to ascertain the involvement of lipid rafts in toxin binding. For example, a 40% reduction in the amount of cholesterol in a CHO cell population failed to abolish internalization of CdtB during intoxication with the *Aa*Cdt [6]. No decrease in cell viability was detected and CdtA remained in association with the cholesterol-depleted membrane. It is important to note that cell death does not typically occur until greater than 50% of total cellular cholesterol is depleted [36]. In a separate study it was found that intoxication of CHO cells with the *Aa*Cdt depended on membrane cholesterol loading of tunicamycin-treated cells [25]. Since tunicamycin inhibits N-linked glycosylation, the implication was that lipid rafts but not a specific glycoprotein receptor was required for *Aa*Cdt binding. In that same study intoxication of CHO cells with *Cj*Cdt was not dependent on cholesterol membrane loading. However, in a different study cholesterol depletion of CHO cells resulted in reduced intoxication with *Cj*Cdt [37]. One possible reason for these contrasting results is that although the CRAC consensus sequence, (L/V)*X*_1-5_Y*X*_1-5_(R/K) is present in a wide variety of proteins it may only be functional in a subset of these proteins. The interaction of the CRAC sequence with cholesterol may be affected by its precise location within the protein, by a specific protein conformation or the microenvironment [38]. These factors could account for the variability observed when examining the interactions of different Cdts with various target cells.

Sphingomyelin appears to accumulate along with cholesterol in lipid rafts [32]. Interestingly, it was found that the *Ec*Cdt, *Aa*Cdt, *Hd*Cdt and *Cj*Cdt required sphingomyelin synthase 1 (SGMS1) for intoxication using the KBM7 gene inactivation screening method [27]. 

In summary, it is possible that the Cdt recognizes susceptible target cells by binding to a specific receptor in the plasma membrane. This process may involve a binding site located within a groove formed between CdtA and CdtC in the holotoxin. A domain, enriched in reactive heterocyclic and aromatic amino acids on the surface of CdtA and adjacent to the groove may tightly anchor the holotoxin on the cell surface. As in the case of other AB toxins, binding may take place at areas of the membrane populated by lipid rafts. A specific receptor could be concentrated in these areas. In addition, a CRAC motif in CdtC may enhance binding of the holotoxin to areas of the membrane enriched for lipid rafts. Therefore, CdtA and CdtC may act cooperatively in toxin binding.

### 3.2. Step 2—Endocytosis

Targeting of pathways or components in the cytosol is a relatively common virulence property of the AB toxins. There are two main types of cytosolic entry: (i) directly from early or late endosomes in response to low pH and (ii) from the ER after transport from the Golgi apparatus by way of endosomes [39]. The Cdt takes the latter route but ends up in the nucleus rather than the cytosol. 

The first important event in intoxication is transport of the relatively large, hydrophilic toxin across the hydrophobic plasma membrane. This process, known as endocytosis, is a general mechanism for the uptake of proteins in all cells. Endocytosis can be aided by the protein clathrin. Clathrin-coated pits reside in the plasma membrane and can facilitate, through ligand-receptor interactions, the concentration of proteins destined for uptake. Shiga and anthrax toxins use clathrin-dependent, receptor-mediated endocytosis to enter target cells [40,41]. Ricin, on the other hand, appears to dissociate from the receptor as the holotoxin enters clathrin-coated pits. However, the uptake process is thought to proceed by a clathrin-independent mechanism of endocytosis [42]. Lipid rafts may also facilitate concentration of the holotoxin in clathrin-coated pits or caveolae (non-clathrin-coated membrane “buds”) during the formation of endosomes. This process can also be either receptor-dependent or independent. 

**Figure 2:**
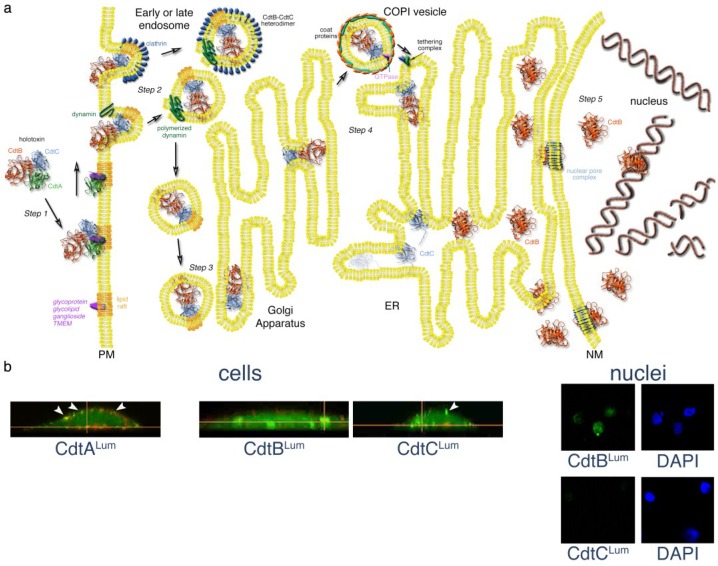
Mechanistic model of Cdt trafficking in mammalian cells. (**a**) After binding to a putative receptor concentrated in membrane lipid rafts, the CdtA subunit remains on the surface. A CdtB-CdtC heterodimer enters the cell by either a clathrin-dependent or independent, receptor-independent endocytosis. Endosomal vesicles are formed by the polymerization of dynamin. Either early or late endosomes deliver the CdtB-CdtC complex to the Golgi. CdtB, the enzymatically-active subunit of the toxin, is moved from the Golgi to the ER by retrograde transport. COPI vesicles, originating from Golgi cisternae, encase the cargo by a process involving the binding of a small GTPase to the coat proteins. The COPI vesicles, directed to the ER by a tethering complex, fuse with the ER membrane. The CdtC protein is degraded in the ER. However, CdtB is translocated from the ER by either an endoplasmic reticulum-associated degradation (ERAD) or non-ERAD pathway. CdtB most likely enters the nucleus through a nuclear pore complex that recognizes a NLS sequence in the protein. However, it is possible that CdtB first enters the cytosol from the ER and then crosses the nuclear membrane through a nuclear pore complex. CdtB binds to DNA in the nucleus and initiates DNA damage by introducing single-and double-strand breaks. Details of the model are presented in the text. Elements of this model were modified from those presented in [48,49,50,51,52]; (**b**) Detection of the *Aa*Cdt subunits during intoxication using a fluorescein arsenical hairpin binding (FlAsH) dye technology and live-cell imaging. *Aa*CdtA was detected only on the CHO cell surface, *Aa*CdtC was present on both the cell surface (arrows) and inside the cells and *Aa*CdtB was found inside the cells and in isolated nuclei. Cells were costained with Lumio™ Green and WGA-Alexa Fluor 555 (red). Nuclei were co-stained with Lumio and DAPI (4',6-diamidino-2-phenylindole). Details of the experiment are provided in the text and in [6].

The Cdt may represent yet another variation of the uptake process by leaving its B1 chain or receptor-binding subunit (CdtA) on the membrane at the surface of the cell (Figure 2a). Retention of a subunit, which is non-essential for biological activity, outside of the cell would be one way to alter the properties of the toxin complex thereby possibly facilitating endocytosis. Evidence of CdtA retention on the cell surface comes from studies in which a fluorescent biarsenic dye tagging technique was used with live-cell imaging to track the location of Cdt subunits labeled with Lumio™ Green (Invitrogen, Grand Island, NY, USA) [6]. In that study, a tetracysteine Lumio™ Green binding sequence (Cys-Cys-Pro-Gly-Cys-Cys) was added to each *Aa*Cdt subunit. Cultures of CHO cells exposed to *Aa*Cdt heterotrimers containing a single modified subunit protein and the corresponding wild-type subunit protein were then labeled with Lumio™ Green. Cells exposed to toxin composed of CdtA^Lum^ and wild-type CdtB and CdtC displayed fluorescence exclusively at the surface of the cells up to 48 h post-intoxication (Figure 2b). Identical results were obtained when the cholesterol content of the cells was unaltered or depleted by treatment with methyl-β-cyclodextrin (MβCD). Retention of CdtA on the cell surface throughout the intoxication process was not surprising due to earlier observations indicating a strong affinity of this subunit for model fucose-containing glycoproteins, such as thyroglobulin, *in vitro* [21,24,30]. Perhaps the aromatic patch region helps trap CdtA on the surface of susceptible cells.

Once the Cdt binds to the plasma membrane the toxin enters cells through the endocytic pathway. It has been reported that the *Hd*Cdt may enter cells by clathrin-dependent endocytosis [43]. Cells in which the formation of clathrin-coated pits was blocked using various treatments, including potassium depletion, were resistant to the toxin. In contrast, cells remained sensitive to the toxin following knock-down of the clathrin gene by RNA interference thus implicating a clathrin-independent process [44]. In addition to a possible role for clathrin during endocytosis of the *Hd*Cdt, the process may also require dynamin II [43,44], a GTPase that controls endocytosis effectors [45]. Dynamin functions by polymerizing to form a narrow constriction leading to scission at the base of the endosomal vesicle [46,47]. 

CHO cells grown in the presence of NH_4_Cl or bafilomycin A1, two inhibitors of endosomal acidification, were resistant to cell cycle arrest when exposed to the *Hd*Cdt and co-localization with the ER was affected [23]. It was found that Rab7 appeared to be important for trafficking of this toxin in HeLa cells and the protein co-localized with Rab9. The RAS-related GTP-binding protein Rab7 participates in the regulation of vesicular transport and is located in late endosomes along with Rab9. A relatively newly discovered inhibitor, EGA (4-bromobenzaldehyde *N*-(2,6-dimethylphenyl)semicarbazone), that blocks protein toxin trafficking by acidified endosomes, affected the ability of the *Hd*Cdt to inhibit the viability of CHO cells [53]. However, EGA failed to alter the effect of *Ec*Cdt on cell viability. Taken together, these findings suggest that the *Hd*Cdt is transported by the late endocytic pathway and the *Ec*Cdt, like some of the other AB toxins (ricin, Shiga and cholera toxin [54,55,56]), may be translocated by early endosomes.

It is likely that a CdtB-CdtC heterodimer is present in endosomes since CdtC was detected on both the surface and inside CHO cells when exposed to *Aa*Cdt composed of CdtA, CdtB and CdtC^Lum^ (Figure 2b) [6]. These observations corroborated the results from an earlier study showing that CdtC could be detected by immunofluorescence microscopy inside the cell [57]. The *Aa*CdtC exhibits much weaker binding *in vitro* to model fucose-containing glycoproteins than CdtA presumably because it lacks a motif comparable to the aromatic patch [30]. If Cdt uptake is receptor- and clathrin-independent then CdtC may play an important role in the endocytosis of CdtB due to binding of a CdtB-CdtC heterodimer to cholesterol in lipid rafts via the CRAC motif.

### 3.3. Step 3—Trafficking to the Golgi Apparatus

Delivery of protein toxins from endosomes to the Golgi apparatus is a common process but is not as clearly understood as endocytosis. Toxin trafficking takes advantage of the secretory pathways of the cell which function in the routine enzymatic modification and distribution of secretory cargo to organelles and the extracellular space. Cargo is either earmarked for degradation in the lysosomal degradation pathway or recycling in the retrograde pathway. The directional movement of cargo from various types of endosomes, such as early, late and recycling, to the Golgi apparatus and *trans*-Golgi network (TGN) has been termed retrograde transport. There are five general groups of retrograde cargoes: (i) cargo sorting receptors; (ii) integral membrane proteases; (iii) SNAREs (soluble *N*-ethylmaleimide-sensitive factor attachment receptors) [48]; (iv) nutrient transporters and (v) some AB toxins [41,58]. 

The endosome contains vacuolar and tubular domains. The tubular endosomal network (TEN) is part of the TGN that represents the last Golgi cisternae and is the primary sorting structure of the retrograde pathway because it contains a relatively high degree of membrane curvature and low lumenal volume [50,59]. These physical properties are ideal for exporting cargo and the splitting off of these tubules forms carriers for retrograde transport. Cargo sorting into the TEN and the microtubule-dependent transport of cargo carriers to the TGN is controlled by retromers that are multi-protein complexes that recognize retrograde cargo [60,61]. Retrograde sorting is facilitated by clathrin and clathrin adapter proteins [62].

As in the case of other toxins little is known about trafficking of the Cdt through the Golgi apparatus. However, there is evidence that at least the *Hd*CdtB subunit undergoes retrograde transport. Based on the results of experiments using Brefeldin A, a well-known inhibitor of the transport of proteins between the ER and the Golgi apparatus, it was found that an intact Golgi complex is required for cell intoxication by the *Hd*Cdt and *Ec*Cdt [23,35]. Brefeldin A blocks the formation of COPI vesicles (see section 3.4). In that same study, *Hd*CdtB, modified to contain a sulfation site, was labeled with ^35^S following the intoxication of cells grown in the presence of Na_2_^35^SO_4_. Sulfation of proteins occurs exclusively in the *trans*-Golgi [63].

### 3.4. Step 4—Passage to the Endoplasmic Reticulum

Membrane traffic between the ER and the Golgi apparatus is bidirectional and is carried out by similar mechanisms [49]. Anterograde transport is the forward transport of newly synthesized proteins and lipids from the ER to the Golgi apparatus towards the plasma membrane. Retrograde transport is the trafficking of proteins from the Golgi back to the ER away from the plasma membrane. There is evidence to indicate that the Golgi apparatus contains 3–4 compartments, composed of multiple cisternae and an ER-Golgi intermediate compartment (ERGIC), in mammalian cells [35,38]. The current model of anterograde trafficking through the Golgi is known as cisternal maturation. In this model *cis*-Golgi cisternae form spontaneously and then develop into *trans*-Golgi cisternae while transporting cargo. The *trans*-Golgi cisternae disintegrate into transport carriers at the TGN or final stage of maturation. In the first stage, COPII (coat protein) vesicles bud from the ER and fuse together to create new *cis*-Golgi cisternae. 

In retrograde transport, COPIa/COPI vesicles, bud from these new *cis*-Golgi cisternae to recycle trafficking proteins from the ERGIC back to the ER. COPI is an ADP ribosylation factor (ARF)-dependent adaptor protein that functions in retrograde trafficking. COPI vesicles are formed when a guanine nucleotide exchange factor (GEF) directs the activation of a GTPase in the Arf1/Sar1 family of proteins in the Golgi membrane [52]. The GTPase forms a complex with the coat protein, cargo and a GTPase activating protein (GAP). The addition of a second layer of coat protein to the complex deforms the membrane and controls the size of the transport vesicle. A catalytically inactive protein kinase, Scyl1, appears to form a scaffold for the COPI coat [64]. In mammalian cells the COPI vesicles that are formed in the *cis*-Golgi most likely use dynein for transportation on microtubules toward the ER [65]. The vesicles are released from the membrane by fission. Typically, retrograde transport is used to recycle proteins that serve as ER export factors, *cis*-Golgi proteins and some ER-resident proteins back to the ER for another round of transport. Sorting signals characterized by the sequences KDEL and KKXX mediate retrograde transport by binding to COPI initiating vesicle formation [66]. This process is exploited by some AB toxins, such as cholera and *Pseudomonas* exotoxin A, to reach the ER for translocation to the cytosol [42]. 

In order for the transport vesicle to deliver its target to the ER it must first be uncoated. The timing and control of coat disassembly have not been well worked out. A tethering complex, DSL1 or syntaxin 18 complex interacts with the COPI vesicle in the cytoplasm or on the ER membrane [52]. This tethering complex initiates a defined series of events that results in fusion of the vesicle with the ER. An activated Rab protein is thought to anchor the tether to the ER membrane and components of the DSL1 complex may facilitate uncoating of the vesicle. Uncoating exposes the v-SNAREs allowing interaction with the t-SNAREs on the ER membrane [48]. This specific interaction may dictate the direction of the transport process since it has been found that Rab proteins participate in the movement of transport carriers along the actin or microtubule cytoskeleton [67,68].

There is also evidence for COPI-independent transport from the Golgi to the ER which may explain why some AB toxins such as ricin and Shiga toxin that lack the KDEL or KKXX motif still traffic to the ER on their way to the cytoplasm [69]. That study indicated that Shiga toxin appears to require Rab6A to enter the ER. However, the precise steps remain to be worked out since there is evidence that Rab6 plays a role in both COPI-dependent and independent mechanisms. The composition of the vesicle coat in this mechanism has not been identified. Ricin does not appear to require Rab6A and may enter the cytoplasm by a Rab- and COPI-independent process [70].

The A chain of AB toxins that have targets in the cytoplasm exit the ER by an endoplasmic-reticulum-associated protein degradation (ERAD) pathway. This pathway generally targets terminally misfolded proteins for ubiquitin-dependent degradation that must take place in the cytosol in proteosomes. In the case of AB toxins, translocation by the ERAD pathway occurs when thermal instability of the A chain, upon dissociation, results in an unfolded conformation [71,72]. The absence of lysine residues in the A chain may protect the translocated protein from ubiquitin-dependent degradation. Possible involvement of the ERAD pathway in the trafficking of some AB toxins from the ER to the cytosol is supported by the observation that CHO cells resistant to ricin and *Pseudomonas* exotoxin A were also partially resistant to cholera toxin [73]. Recently, proteins or protein complexes possibly involved in the retrograde trafficking of the *Pseudomonas* exotoxin A from the Golgi to the ER were identified using a haploid genetic screening technique [74]. These proteins included the tethering complex GARP (Golgi-associated retrograde protein), KDELR1 (KDEL receptor), AP1M1 (a clathrin-interacting protein involved in vesicle trafficking), SCFD1 (Sec1 family domain-containing protein 1), OSTC (oligosaccharyltransferase complex subunit) and GPR107 (an orphan G-protein-coupled receptor). Among these proteins, only GRP107 was detected when the same technique was used to screen for trafficking components for the *Cj*Cdt [27,74]. In contrast, the *Aa*Cdt, *Ec*Cdt and *Hd*Cdt killed GRP107 null cells.

The A chain (CdtB) of the *Hd*Cdt does not appear to be translocated from the ER by the typical ERAD pathway since CHO cells, selected for resistance to *Pseudomonas* exotoxin A and cholera toxin, were sensitive to the Cdt [35]. This may be in part because the primary activity of CdtB is that of a nuclease that enters the nucleoplasm rather than the cytoplasm. In addition, the *Hd*CdtB is heat stable, maintaining its folded conformation at a temperature and buffering conditions that are similar to those of the ER, and does not appear to unfold prior to exiting this organelle. Similarly, the plasmid-encoded toxin (Pet), a non-AB toxin, from enteroaggregative *E. coli* is a thermally stable protein and appears to enter the cytosol by an ERAD-type translocation based on the partial exposure of hydrophobic residues instead of spontaneously unfolding [75]. 

More recently, evidence demonstrating that three basic components of the ERAD system, Derl2, Hrd1 and p97, are required for intoxication of CHO and HeLa cells by the *Hd*Cdt, *Ec*Cdt, *Aa*Cdt and *Cj*Cdt [76]. Derl2, a member of the Derlin family of proteins may function by forming a channel for the retrotranslocation of misfolded proteins to the cytosol. Hrd1 is involved in the retrograde transport of unfolded proteins to the cytosol and p97 has been implicated in the retrotranslocation of proteins by the ubiquitin-proteasome pathway. Dependency on these ERAD proteins for translocation of the four Cdts examined appears to diminish consistent with the evolutionary divergence of the species-specific Cdts. These findings contrasted those supporting an ERAD-independent model [26].

Retrograde translocation of the *Hd*CdtB was indicated when this protein became labeled when HeLa cells were grown in the presence of []-mannose [35]. *N*-linked glycosylation of proteins is carried out post-translationally in the ER. As pointed out by Guerra and coworkers [77], it is possible that the *Hd*CdtB is first translocated from the ER to the cytosol where it enters the nucleus through a nuclear pore complex. Some toxins, such as cholera toxin, that have the AB_5_ configuration are translocated intact from the Golgi complex to the ER. In the ER the toxin dissociates into the A and B moieties by reduction of the disulfide bridges linking the A1 and A2 polypeptides of the A chain [78,79]. The A1 chain unfolds by interacting with a chaperone and is delivered to the cytosol. Although the detailed steps in this process have not been elucidated, the results of a genome-wide siRNA screen found that specific host factors are required for ricin and Shiga-toxin that may indicate translocation to the cytosol by a separate mechanism. Based on studies with the *Aa*Cdt, it is possible that the CdtC subunit is delivered as a heterodimer with CdtB to the Golgi apparatus and ER since each of these proteins was detected by Lumio™ Green labeling inside CHO cells that had been exposed separately to toxin reconstituted with either CdtB^Lum^ or CdtC^Lum^ [6]. However, there is no direct experimental evidence to confirm that both of these proteins were present within the Golgi complex or ER. It is possible that the *Aa*CdtC undergoes degradation by the standard ERAD pathway. The fact that intoxicated CHO cells in these experiments succumbed to cell cycle-arrest was indirect proof that the *Aa*CdtB subunit undergoes retrograde transport through the Golgi apparatus and ER on its way to the nucleus.

### 3.5. Step 5—Entering the Nucleus

The Cdt is the only AB toxin, identified to date, that traffics to the nucleus. Many of the other members of this toxin superfamily have a cytosolic target [1]. Translocation of CdtB to the nucleus in a variety of Cdt-sensitive cells seems to indicate that DNA nicking or cutting is the primary mode of action of the toxin. Even though there is evidence that *Aa*CdtC enters the cell along with *Aa*CdtB, the former subunit has not been located in the nucleus. *Aa*CdtC^Lum^ was not found in nuclei isolated from CHO cells intoxicated with toxin comprised of this subunit and wild-type *Aa*CdtA and *Aa*CdtB (Figure 2b) [6]. In contrast, *Aa*CdtB^Lum^ was readily detected in the nucleus when cells were exposed to *Aa*CdtAB^Lum^C. *Aa*CdtB^Lum^ appeared in the nucleus in as little as 4.5 hours post-intoxication. Assays used in several other studies of the translocation of the *Ec*Cdt and *Hd*Cdt found that CdtB reaches the nucleus very rapidly, in some instances in as little as 60 minutes [23,76]. Other studies that examined translocation of the various species-specific CdtBs to the nucleus did not report the presence of CdtC (summarized in [44]). Microinjection of *Cj*CdtB alone into the cytoplasm of COS-1 and REF52 cells resulted in cell cycle arrest within 4 hours and established that *Cj*CdtC was not required to obtain inhibitory activity [80]. Taken together these data indicate that the final step in the trafficking of the Cdt is the translocation of only the CdtB subunit to the nucleus.

The identification of a nuclear localization signal (NLS) sequence in CdtB was the first indication that the A chain of an AB toxin is capable of translocating to the nucleus. NLS are ubiquitous in eukaryotic cytoplasmic proteins that need to enter the nucleus. Typical NLS sequences are: RKRKL, KRPAAIKKAGQAKKKK, RRRHSDENDGGQPHKRRK and NQSSNFGPMKGGNFGGRSSGPYGGGGQYFAKPRNQGGY [81]. Some viral proteins such as the simian virus (SV40) large T-antigen have taken advantage of the NLS strategy [82]. This NLS sequence, PKKKRKV, was the prototype but many sequences discovered since are more complex. However, no consensus sequence has been identified [83]. In one model of NLS sequence function a class of proteins known as importins recognize the NLS and carry the targeted protein through a nuclear pore [84]. The NLS-containing protein-importin complex is dissociated by GTP-bound Ran which releases the target protein in the nucleus. Ran (RAS-related nuclear protein) is a small G protein required for the translocation of RNA and proteins through the nuclear pore complex and also may be involved in the control of DNA synthesis and cell cycle progression. The Ran-GTP/importin complex is returned to the cytoplasm and a RanGAP1 (GTPase-activating protein) recycles the free importin.

Even though a NLS sequence appears to be essential for translocation of CdtB across the nuclear membrane, several complications have made it difficult to assess the molecular details of the process. It has been reported that the NLS is located in the amino-terminal region of the *Aa*CdtB (amino acids 48-124) [85] and is composed of two regions (amino acids 195-210 and 253-268) in the carboxy-terminal region of *Ec*IICdtB [86]. Mutagenesis experiments indicated that monopartite or bipartite arginine and/or lysine residues, necessary for classical NLS function, might not be required by the *Aa*CdtB and *Ec*IICdtB NLS [6,85,86]. Therefore, the CdtB NLS is considered to be an atypical sequence. It is also not known if the NLS functions in the movement of proteins from the ER to the nucleus since is possible that the NLS could direct the CdtB into the nucleus from the cytoplasm if this protein is released from the ER by an unidentified non-ERAD pathway.

## 4. Conclusions

Retrograde transport of toxins via the Golgi apparatus and ER requires a complex cascade of supporting protein chaperones, protein complexes, small GTPases and unidentified components. The process is complicated further by observations that various toxins have evolved to hijack different components of the TGN and ER protein processing machinery. Although significant progress has been made in understanding the entry of some AB toxins such as ricin, Shiga toxin, cholera toxin, and *Pseudomonas* exotoxin A, the study of Cdt translocation is in its infancy. Part of the challenge in elucidating the precise molecular details of Cdt trafficking is the unique aspects of this subclass of AB toxins. Unique properties exhibited by some or all of the species-specific Cdts are translocation by a late endosomal pathway, ER processing possibly by an atypical non-ERAD pathway and directed transport to the nucleus due to the presence of an atypical NLS sequence. Conclusions based on the results of multiple independent investigations suggest that the various species-specific Cdts require different membrane receptors, interact differently with membrane lipids, exhibit different target cell specificities and inhibit different cell pathways thereby further complicating efforts to elucidate the details of toxin trafficking. It is well established that toxins supply versatile tools for probing the complex functions of the Golgi apparatus and ER [87]. It is predicted that the unique properties of the Cdt will provide even more versatility in studying the workings of these cell structures, may lead to the discovery of new translocation components and pathways and may extend the utility of this subclass of AB toxins as tools for developing novel therapeutic interventions for bacterial infections.
